# Prediction of Two-Dimensional Janus Transition-Metal Chalcogenides: Robust Ferromagnetic Semiconductor with High Curie Temperature

**DOI:** 10.3390/molecules29163915

**Published:** 2024-08-19

**Authors:** Zijin Wang, Ali Hamza Qureshi, Yuanyuan Duan, Yujie Liu, Yanbiao Wang, Jun Zhu, Jinlian Lu, Tianxia Guo, Yongjun Liu, Xiuyun Zhang

**Affiliations:** 1 College of Physics Science and Technology, Yangzhou University, Yangzhou 225002, China; 210903135@stu.yzu.edu.cn (Z.W.); gtx990920@outlook.com (T.G.);; 2 Department of Fundamental Courses, Wuxi Institute of Technology, Wuxi 214121, China; 3 Department of Physics, Yancheng Institute of Technology, Yancheng 224051, China; ljlmalou@163.com

**Keywords:** ferromagnetic, semiconductor, high Curie temperature, spintronics

## Abstract

Two-dimensional (2D) ferromagnetic semiconductors (FM SCs) provide an ideal platform for the development of quantum information technology in nanoscale devices. However, many developed 2D FM materials present a very low Curie temperature (T_C_), greatly limiting their application in spintronic devices. In this work, we predict two stable 2D transition metal chalcogenides, V_3_Se_3_X_2_ (X = S, Te) monolayers, by using first-principles calculations. Our results show that the V_3_Se_3_Te_2_ monolayer is a robust bipolar magnetic SC with a moderate bandgap of 0.53 eV, while V_3_Se_3_S_2_ is a direct band-gap FM SC with a bandgap of 0.59 eV. Interestingly, the ferromagnetisms of both monolayers are robust due to the V–S/Se/Te–V superexchange interaction, and T_C_s are about 406 K and 301 K, respectively. Applying biaxial strains, the FM SC to antiferromagnetic (AFM) SC transition is revealed at 5% and 3% of biaxial tensile strain. In addition, their high mechanical, dynamical, and thermal stabilities are further verified by phonon dispersion calculations and ab initio molecular dynamics (AIMD) calculations. Their outstanding attributes render the V_3_Se_3_Y_2_ (Y = S, Te) monolayers promising candidates as 2D FM SCs for a wide range of applications.

## 1. Introduction 

With the development of device minimization processing, various two-dimensional (2D) semiconductors (SC) with large surface-to-volume ratios and intriguing electronic properties have attracted intensive attention [1,2,3,4,5,6,7,8]. Among them, the emergence of 2D ferromagnetic semiconductors (FM SCs) has drawn particular interest by combining the complementary function of SCs and FM materials, allowing the coupling and dependent control of electron charge and spin [9,10,11]. Compared with conventional electronics based on electron charge degree of freedom [12,13,14], 2D FM SCs show advantages of high energy-efficient performance, faster information operation, non-volatile data storage, etc. Unfortunately, most 2D semiconducting materials are found to be nonmagnetic due to their incompatibility between semi-conductivity and magnetism [15]. In addition, a long-range magnetic ordering in magnetism is difficult to maintain because of thermal agitation, according to the Mermin–Wagner theorem [16]. These challenges largely limit the widespread applications of 2D FM SC-based spintronics devices.

The experimental breakthroughs in 2D FM SCs were the successful preparation of the CrI_3_ monolayer and Cr_2_Ge_2_Te_6_ bilayer in 2017 [17,18]. Inspired by these achievements, a number of 2D FM SCs were fabricated and predicted by experimental and theoretical researchers, including transition-metal (TM) halides [17], TM dichalcogenides (TMDs) [19], TM chalcogenide-halide Janus complexes [20], and their various derivatives [21]. Despite the progress, most of these FM SCs possess lower than room temperature T_C_. For example, the T_C_s of the CrI_3_ monolayer [22] and the Cr_2_Ge_2_Te_6_ bilayer [23] are as low as 45 K and 63 K, respectively, which greatly limits their practical applications. Alternatively, 2D Janus TMDCs with hetero chalcogenide elements are found to show novel electronic and magnetic properties due to their broken structural symmetry [24]. In addition, robust ferromagnetic and half-metallic characters are identified for Janus FeXY (X, Y = Cl, Br, and I, X ≠ Y) monolayers [25]. The 2D VSSe was found to be a highly stable room-temperature FM SC with valley polarization feature [20]. The 2D MnSSe was revealed to be high T_C_ FM half-metal, in which the magnetization easy axis can be tuned by hole and electron doping [26]. Research on the Janus structure then reached a high point, aiming to explore materials with better performance.

In this work, by using the first-principles method, we predict two high-temperature 2D Janus FM SCs, namely, V_3_Se_3_X_2_ (X = S, Te) monolayers. Both systems are predicted to be thermodynamically stable. Interestingly, the ferromagnets of both structures are found to be robust based on the V–S/Se/Te–V superexchange interaction and their T_C_s are about 406 K and 301 K for V_3_Se_3_S_2_ and V_3_Se_3_Te_2_, respectively. Moreover, the electronic and magnetic properties of both systems can be flexibly modulated under external in-plane biaxial strains.

## 2. Results and Discussion

**Figure 1:**
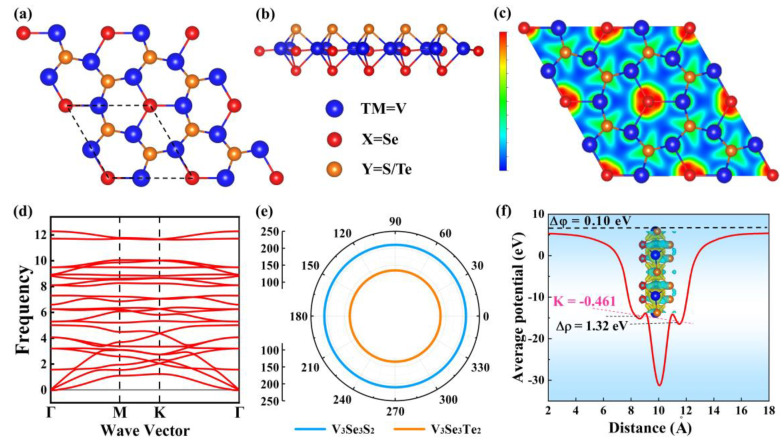
The (**a**) top view and (**b**) side view of the V_3_Se_3_S_2_ (V_3_Se_3_Te_2_) monolayer. (**c**) The calculated electron localization function (ELF) of the V_3_Se_3_S_2_ monolayer for the (001) plane. (**d**) Phonon dispersion spectrum of V_3_Se_3_S_2_ monolayer. (**e**) Young’s modulus of V_3_Se_3_S_2_ (Blue) and V_3_Se_3_Te_2_ (Orange). (**f**) Average electrostatic potential of the V_3_Se_3_S_2_ monolayer.

Figure 1a,b presents the crystal structure of monolayer V_3_Se_3_X_2_ (X = S, Te), which is a three-atomic-layer thickness incorporating V, Se, and X atoms. Their space group is *P*3*m*1, and the lattice constants are 5.76 Å and 5.95 Å for the V_3_Se_3_S_2_ and V_3_Se_3_Te_2_ monolayers, respectively, in which, the V-V and the V-Se bond lengths in the middle plane are around 3.00~3.12 Å and 2.44~2.47 Å, and the V-S/Te and V-Se bond lengths out of the middle plane are around 2.31~2.67 Å. To address the structural stability and experimental feasibility of both V_3_Se_3_X_2_ monolayers, we have calculated their cohesive energies (*E_coh_*) and formation energies (*E_f_*) based on the following equations:

(1) $$E_{f}=\frac{E_{V3Se3X2}-(3\mu_{V}+3\mu_{Se}+2\mu_{X})}{n}$$

(2) $$E_{coh}=\frac{E_{V3Se3X2}-\left(3E_{V}+3E_{Se}+2E_{X}\right)}{n}$$

Here, *E_V3Se3X2_*, *μ_V_*, *μ_Se_*, and *μ_Y_* are the total energy of V_3_Se_3_X_2_ monolayers, and the energies of V, Se, and S/Te atoms in their bulk crystals, respectively. *E_V_*, *E_Se_*, and *E_X_* are the energies of isolated V, Se, and S/Te atoms. The calculated *E_coh_*s of both systems are −4.96 eV/atom and −4.52 eV/atom, respectively, comparable to that of the MoS_2_ (−4.53 eV/atom) and MoSe_2_ (−5.07 eV/atom) monolayers [27]. In addition, the *E_f_*s are −0.99 eV and −0.83 eV for V_3_Se_3_S_2_ and V_3_Se_3_Te_2_, respectively; the negative values ensure their fabrication feasibility in the experiment. Figure 1c shows the electron localization function (ELF) of the V_3_Se_3_S_2_ monolayer, from which we can see that the electrons are mainly delocalized around each atom, indicating their obvious ionic characters. The average electrostatic potential and charge density difference (CDD) plot of the V_3_Se_3_S_2_ monolayer is shown in Figure 1f. Clearly, the electrostatic potential of the S atom layer is greater than that of Se atom layer, due to the fact that the electronegativity of the S atom is greater than that of the Se atom. As a result, more electrons are found to be transferred from V atoms to S atoms than Se atoms.

The phonon spectra of the V_3_Se_3_S_2_ and V_3_Se_3_Te_2_ monolayers are illustrated in Figure 1d and Figure S1 in the Supporting Information (SI); the absence of imaginary frequencies in the Brillouin zone confirms the dynamical stabilities of both systems. In addition, the thermal stability of the V_3_Se_3_X_2_ (X = S, Te) monolayers is assessed by AIMD simulations at 300 K for 6 ps. As illustrated in Figure S2, the energies of the V_3_Se_3_X_2_ (X = S, Te) monolayers fluctuate within a narrow range during the simulation, and the final snapshot demonstrates that the structures remain well-preserved, indicating excellent thermal stability. Additionally, the mechanical stabilities for both systems are evaluated by calculating Young’s modulus and the Poisson ratio. The calculated elastic constants for the V_3_Se_3_S_2_/V_3_Se_3_Te_2_ monolayers are C_11_ = 247.90/193.33 N/m, C_12_ = 96.92/106.56 N/m, C_22_ = 247.90/193.33 N/m, and C_66_ = 75.49/43.38 N/m, respectively, which satisfy the Born stability criterion (C_11_, C_66_ > 0, C_11_–C_22_ > 0 and C_11_ + 2C_12_ > 0), indicating their mechanical stabilities. Young’s modulus and Poisson’s ratio as a function of *θ* angle (the angle between a certain direction and *x*-axis) are shown in Figure S3 for both the monolayers. It is found that Young’s modulus for the V_3_Se_3_S_2_ and V_3_Se_3_Te_2_ monolayers are isotropic with Young’s modulus for 210.01 N/m^2^ and 134.59 N/m^2^, respectively; in contrast, Poisson’s ratio for both systems is anisotropic.

**Figure 2:**
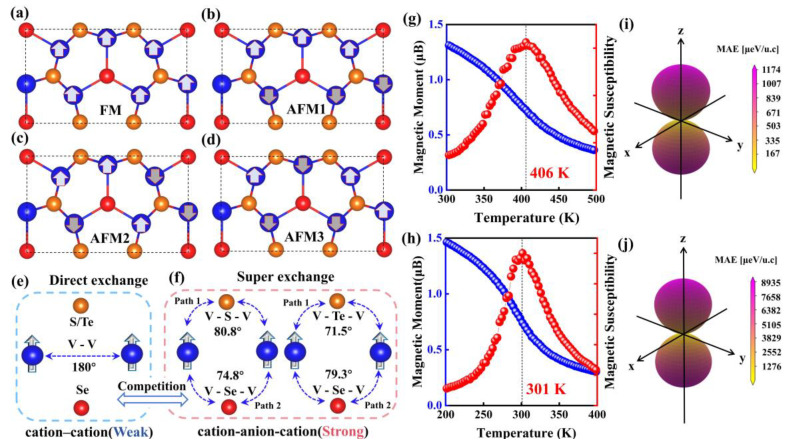
(**a**–**d**) FM and AFM orderings. (**e**,**f**) illustrations of the V–V direct-exchange, V–S/Se/Te–V superexchange. The magnetic moment and Magnetic susceptibility of the V atom as a function of temperature for the (**g**) V_3_Se_3_S_2_ and (**h**) V_3_Se_3_Te_2_ monolayer, respectively. The magnetocrystalline anisotropy energy (MAE) for (**i**) V_3_Se_3_S_2_ and (**j**) V_3_Se_3_Te_2_ monolayer.

To determine the magnetic ground states of both V_3_Se_3_X_2_ (X = S, Te) monolayers, four magnetic configurations, namely, FM, AFM1, AFM2, and AFM3 are considered, as depicted in Figure 2a–d. Upon comparison, the FM orderings are the favored magnetic states for all the structures, which are lower in energy than AFM1, AFM2, and AFM3 by 0.080, 0.064, and 0.044 eV/atom, respectively, for V_3_Se_3_S_2_ and by 0.064, 0.046, and 0.026 eV/atom for V_3_Se_3_Te_2_. The FM ordering in both V_3_Se_3_S_2_ and V_3_Se_3_Te_2_ monolayers originate from the competition between direct and superexchange interactions (see Figure 2e,f). Direct exchange interactions are based on the overlap of wave functions between two neighboring magnetic ions, which usually leads to AFM coupling. [28] As the distance between two neighboring V atoms is as long as 3.00 and 3.12 Å, longer than that in bulk V (2.62 Å), the direct exchange interactions in both systems are very weak. On the other hand, the superexchange paths are mediated by X or Y atoms (Path 1, 2), as shown in Figure 2f, in which the V-Se-V/V-Y-V angles are around 80° and 75°, close to 90°. Therefore, the superexchange interactions determine the FM couplings of magnetic ions according to the Goodenough–Kanamori–Anderson (GKA) rule. Considering the Coulomb exchange interaction between S/Te *p* orbitals and the fact that the bond angle of V(I)–S/Te–V(II) is 80.8° in V_3_Se_3_S_2_ and 71.5° in V_3_Se_3_Te_2_ (relatively close to 90°), it can be inferred that the superexchange interaction brings a strong FM order [29,30]. As shown in Figure 2f, there are two kinds of V–Se–V/V-Y-V superexchange coupling pathways in each monolayer (Paths 1, 2) due to the presence of Se and Y elements. In V_3_Se_3_S_2_, the electrons mainly hop between V(I) *d*_x2_/V(II) *d*_z2_ via S *p* states, as shown in Figure 2a,b, which implies that electrons favor path 1. In V_3_Se_3_Te_2_, the electrons mainly hop between V(I) *d*_x2_/V(II) *d*_z2_ via Te *p* states, as shown in Figure 2c,d, implying path 1 is again more favored.

**Figure 3:**
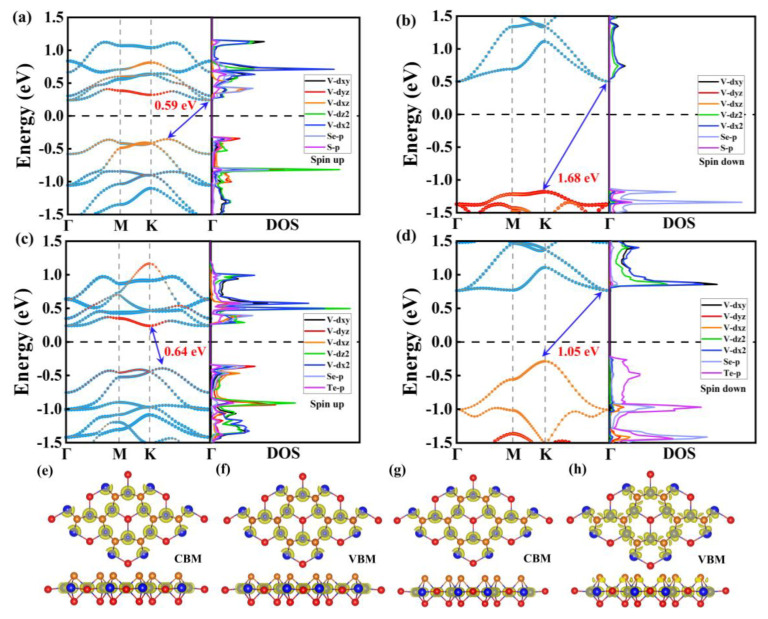
Bands and DOS of SL V_3_Se_3_S_2_ in (**a**) spin-up and (**b**) spin-down channels. Bands and DOS of SL V_3_Se_3_Te_2_ in (**c**) spin-up and (**d**) spin-down channels. (**e**) CBM and (**f**) VBM of SL V_3_Se_3_S_2_. (**g**) CBM and (**h**) VBM of SL V_3_Se_3_Te_2_.

To further investigate the electronic properties of both V_3_Se_3_X_2_ monolayers, we have calculated the spin-resolved band structures and density of states (DOS) (Figure 3a–d). Notably, the V_3_Se_3_S_2_ monolayer is an FM SC with a direct bandgap of 0.59 eV, while the V_3_Se_3_Te_2_ monolayer exhibits an FM SC character with an indirect gap of 0.53 eV. Of particular interest, the conduction band minimum (CBM) and the valence band maximum (VBM) of the V_3_Se_3_Te_2_ monolayer are fully spin-polarized and occupied with opposite spin electrons, indicating a typical bipolar magnetic semiconductor (BMS) feature, as shown in Figure 3c,d [31,32,33]. As can be seen from the DOS in Figure 3a,b, the VBM and CBM are both mainly contributed to by the *d*_x2_–*d*_z2_ orbits of the V atom and p orbits of S and Te, respectively.

Generally, the Curie temperature (T_C_) also strongly correlates with the magnetic anisotropic energy (MAE), while it is also the key to the practical application of materials in spintronics. We use the classic Heisenberg model to investigate the magnetic couplings [34,35], the spin Hamiltonian can be considered as

(3) $$H=E_{0}-J_{1}\sum_{i,j}\;S_{i}S_{j}-J_{2}\sum_{i,k}\;S_{i}S_{k}-J_{3}\sum_{i,u}\;S_{i}S_{u}-A(S_{i}^{z}{)}^{2}$$

Here, *E*_0_ is the energy without considering the interaction between magnetic atoms V. *J*_1_, *J*_2_, and *J*_3_ represent the first-nearest, second-nearest, and third-nearest spin exchange coupling parameters. *S_i_*, *S_j_*, *S_k_*, *and S_u_* stand for the spin vector, and *S_i_^z^* is the spin component. *A* is an anisotropy energy parameter [28,32,36]. The values of *J*_1_, *J*_2_, and *J*_3_ are computed to be 14.14, 36.16, and 5.14 meV for V_3_Se_3_S_2_ and 3.80 meV, 38.76 meV, and 5.98 meV for V_3_Se_3_Te_2_, respectively. A 10 × 10 supercell and periodic boundary conditions are adopted for the MC simulation. The magnetic moment and magnetic susceptibility are mapped out as functions of temperature in Figure 2g,h. The estimated transition temperatures for SL V_3_Se_3_S_2_ and V_3_Se_3_Te_2_ monolayers are ~406 K and ~301 K, respectively, which are higher than those of CrX_3_ (X = Cl, Br, I) monolayers [18,37,38] and the Cr_2_Ge_2_Te_6_ bilayer [17]. The main reason for a relatively high T_C_ is the large positive magnetic exchange parameter and large MAE, which is also inseparable from the strong hybridization between V and the Te/Se/S atom.

**Figure 4:**
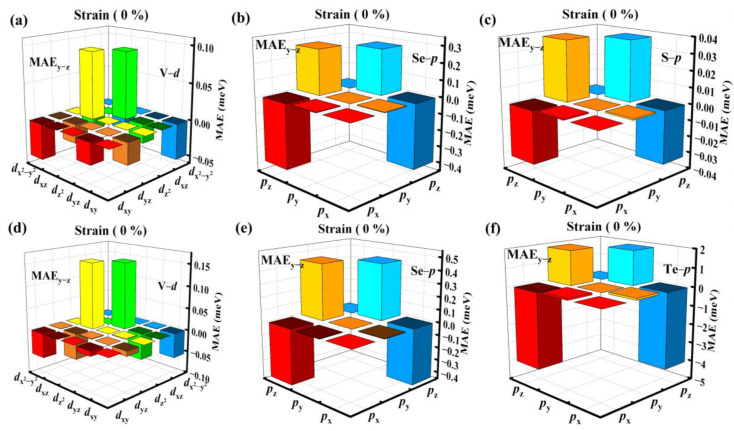
Orbital resolved MAE for (**a**–**c**) V_3_Se_3_S_2_ and (**d**–**f**) V_3_Se_3_Te_2_, respectively.

The MAEs are calculated to check the magnetic easy axis of each system as E_MAE_ = |E_in-plane_ − E_out-of-plane_|, which is defined as the difference between the energy of in-plane and out-plane magnetization directions. As shown in Figure 2e,f, it is found that the MAE values of V_3_Se_3_S_2_ and V_3_Se_3_Te_2_ are 196 and 1489 μeV per V atom, larger than that of CrS_2_ (88.5 μeV per Cr) and CrSe_2_ (664.0 μeV per Cr) [13]. Their magnetization directions are both in-plane, such large MAEs have rarely been observed in 2D magnetic materials. To further analyze the origin of such large MAEs, orbital resolved MAEs are displayed in Figure 4a–f, illustrating that for the V_3_Se_3_S_2_ monolayer, the coupling strength between the *p*_y_ and *p*_z_ orbitals of the Se atom is the largest, next is the coupling between the *d*_xz_ and V *d*_z2_ orbitals of the V atom. It can be seen that the Se *p*_z_ orbital has a big influence on MAE, contributing to the negative part. In addition, the coupling strength between the Se *p*_y_ and Se *p*_z_ orbitals is also strong with a positive value, but slightly weaker than that of the Se *p*_z_ orbitals. For the V_3_Se_3_Te_2_ monolayer, the Te *p*_z_ orbital makes the biggest contribution to the MAE, with the coupling between the Se *p*_y_ and Se *p*_z_ orbitals coming next. As for the V *d* orbitals, it is the coupling of the *d*_z2_ and *d*_xz_ orbitals that dominates, similar to that of the V_3_Se_3_S_2_ monolayer. However, the value is relatively small compared to that of the Se *p* and Te *p* orbitals, making little impact on the total MAE value.

**Figure 5:**
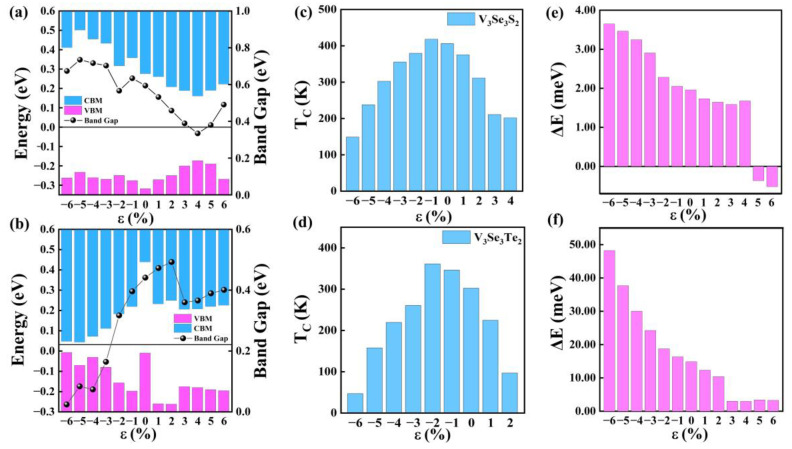
Band alignment scheme VBM of the (**a**) V_3_Se_3_S_2_ monolayer and (**b**) V_3_Se_3_S_2_ monolayer. (**c**,**d**) Curie temperature (T_C_) of the V_3_Se_3_S_2_ and V_3_Se_3_Te_2_ monolayers under different strains. MAE = E_out-plane_ − E_in-plane_ of the (**e**) V_3_Se_3_Te_2_ monolayer and (**f**) V_3_Se_3_Te_2_ monolayer plotted as a function of strain.

Furthermore, the effect of biaxial strains on the electronic and magnetic properties of V_3_Se_3_X_2_ monolayers is explored (see Figures S4 and S5). It is clear that the V_3_Se_3_S_2_ monolayer keeps the FM SC character in the range from −6% compressive strain to 4% tensile strain, and, under −5% and 6% tensile strain, it is changed to be an AFM BMS (see Figure S4). On the other hand, the V_3_Se_3_Te_2_ monolayer transitioned to be an AFM SC under 3% tensile strain (see Figure S5). Moreover, the band gap of the V_3_Se_3_S_2_ monolayer decreases with the external strains, ranging from −6% to 4%, and goes up slightly under tensile strains, from 4% to 6%, where the VBM experiences a little fluctuation and the CBM gets closer to the Fermi surface (see Figure 5a and Figure S4). For the V_3_Se_3_Te_2_ monolayer, the FM BMS property is maintained under strains from −6% to 2%; remarkably, the V_3_Se_3_Te_2_ monolayer undergoes a FM SC to AFM SC transition under 3% tensile strain (see Figure S5). Unlike the V_3_Se_3_S_2_ monolayer, the band gap of the V_3_Se_3_Te_2_ monolayer increases monotonously with the external strain, where the VBM and CBM get away from the Fermi surface (see Figure 5b).

Finally, the influence of biaxial strains on the Curie temperature (T_C_) of the V_3_Se_3_S_2_ and V_3_Se_3_Te_2_ monolayers is also studied. It is observed that the T_C_s of both materials decrease under tensile strain, while they initially increase and then decrease under compressive strain (see Figure 5c,d). Notably, the T_C_s of the V_3_Se_3_S_2_ and V_3_Se_3_Te_2_ monolayers reach 418 K and 361 K when subjected to −1% and −2% compressive strains, respectively. This behavior demonstrates the tunability of the electronic properties of V_3_Se_3_Y_2_ (Y = S, Te) monolayers through strain engineering, thereby enhancing their potential applications in spintronic devices. Moreover, the variation of the magnetic anisotropy energies (MAE) are analyzed and presented in Figure 5e,f. It is observed that the MAE of V_3_Se_3_S_2_ consistently decreases within the range of −6% to 3% strain and slightly goes up at 4% strain. However, it goes below 0 meV at 5% to 6%, owing to the transition from FM SC to AFM SC. The MAE of V_3_Se_3_Te_2_ experiences a sharper decrease when subjected to strains from −6% to 2% and experiences a sudden decrease upon reaching a tensile strain of 3% due to the phase transition from FM SC to AFM SC.

## 3. Computational Methods

All the calculations are performed via the density functional theory (DFT) as implemented in the Vienna ab initio simulation package (VASP) [39]. The Perdew–Burke–Ernzerhof (PBE) [40] method based on the generalized gradient approximation (GGA) [41,42] is used to treat the exchange-correlation functional, and the electronic interaction is treated by the projector augmented wave (PAW) method [43]. To consider the Coulomb and exchange interaction of V 3d electrons, the PBE+U with U = 3 eV is adopted. The plane-wave energy cutoff is set to 500 eV, and the vacuum layer of 20 Å is employed for the studied systems to eliminate adjacent layer interactions. The geometries are fully relaxed until the force and energy convergence are less than 0.01 eV/Å and 10−6 eV, respectively. A Γ-centered k mesh of 12 × 12 × 1 is used for geometry optimization calculations. The Curie temperature (T_C_) of both monolayers is calculated by using the EspinS package [44], in which 10 × 10 × 1 lattices are adopted in the Monte Carlo (MC) simulations, and the spins are randomly rotated in the space. A 3 × 3 × 1 supercell of the V3Se3S2 and V3Se3Te2 monolayers are used to calculate the phonon dispersion spectrum by the PHONOPY code based on the density functional perturbation theory [45].

## 4. Conclusions

In summary, two types of 2D intrinsic FM SC materials, V_3_Se_3_S_2_ and V_3_Se_3_Te_2_ monolayers, are predicted by density functional theory calculations. Both of them are revealed to have good thermal, dynamic, and mechanical stabilities. Our results show that V_3_Se_3_S_2_ and V_3_Se_3_Te_2_ monolayers show robust ferromagnetism and above room temperature T_C_ of 406 K and 301 K, respectively. The robust ferromagnetic properties are induced by the V–S/Se/Te–V superexchange interaction. V_3_Se_3_S_2_ monolayer is BMS with band gap of 0.59 eV, and V_3_Se_3_Te_2_ monolayer is an indirect band semiconductor with the band gap of 0.53 eV. When biaxial strain is introduced, a FM SC to AFM SC phase transition is found for both systems with the transition point at 5% and 3% tensile strain for the V_3_Se_3_S_2_ and V_3_Se_3_Te_2_ monolayers, respectively. Our results provide an effective method for designing promising candidates for FM SCs, which also provide opportunities for future spintronic research and applications.

## Data Availability

The data presented in this study are available on request from the corresponding author.

## Supplementary Materials

The following supporting information can be downloaded at: https://www.mdpi.com/article/10.3390/molecules29163915/s1, Figure S1: Phonon spectra of (a) V_3_Se_3_S_2_ and (b) V_3_Se_3_Te_2_; Figure S2: MD simulation was performed for geometric snapshot of the (a) V_3_Se_3_S_2_ and (b) V_3_Se_3_Te_2_ monolayer at 300 K for 6 ps; Figure S3: Young’s modules of (a) V_3_Se_3_S_2_ and (b) V3Se3Te2 respectively and Poisson’s ratio of (c) V_3_Se_3_S_2_ and (d) V_3_Se_3_Te_2_; Figure S4: Band structures of V_3_Se_3_S_2_ under biaxial strain from −6%~6%; Figure S5: Band structures of V_3_Se_3_Te_2_ under biaxial strain from −6%.
