# Supplementary material for: Prediction of Two-Dimensional Janus Transition-Metal Chalcogenides: Robust Ferromagnetic Semiconductor with High Curie Temperature
Source: Molecules. 2024 Aug 19;29(16):3915. doi: 10.3390/molecules29163915 (PMC11357067; doi:10.3390/molecules29163915)
Supplement: Supplementary file 1 [file molecules-29-03915-s001.zip › molecules-3135232-supplementary.pdf]

Supporting Information

Prediction of Two-Dimensional Janus Transition Metal Chalcogenides: Robust Ferromagnetic Semiconductor with High Curie Temperature

Zijin Wang,[1] Ali Hamza Qureshi,[1] Yuanyuan Duan,[1] Yujie Liu,[1] Yanbiao Wang,[2*] Jun, Zhu,[1] Jinlian Lu,[3] Tianxia Guo,[1*] Yongjun Liu,[1] Xiuyun Zhang[1*]

[1]College of Physics Science and Technology, Yangzhou University, Yangzhou 225002, China

[2]Department of Fundamental Courses, Wuxi Institute of Technology, Wuxi 214121, China

[3]Department of Physics, Yancheng Institute of Technology, Yancheng, Jiangsu 224051, China.

Email: wangyb@wxit.edu.cn ; xyzhang@yzu.edu.cn

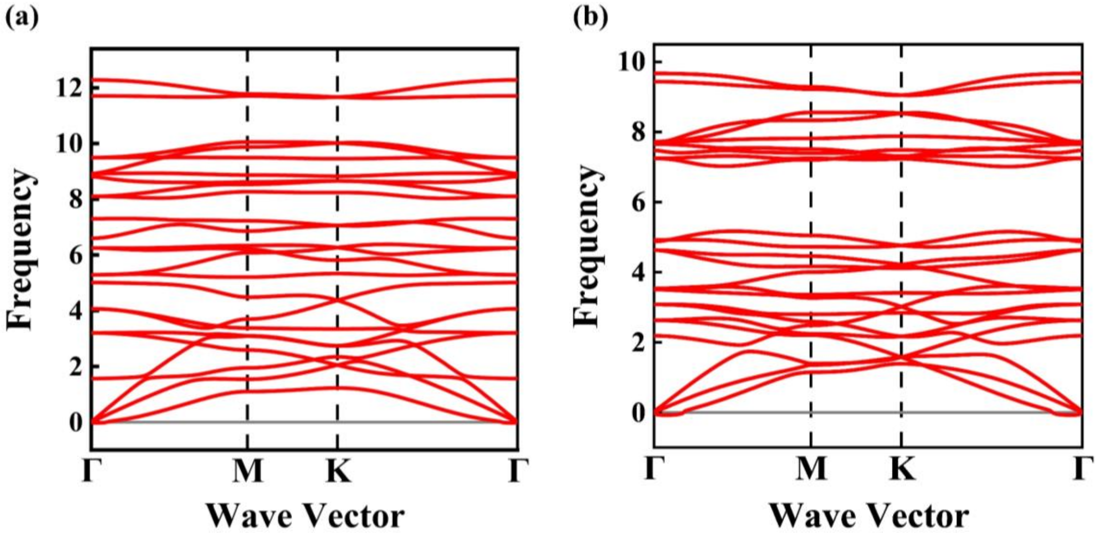

Figure. S1 Phonon spectra of (a) $V_3Se_3S_2$ and (b) $V_3Se_3Te_2$.

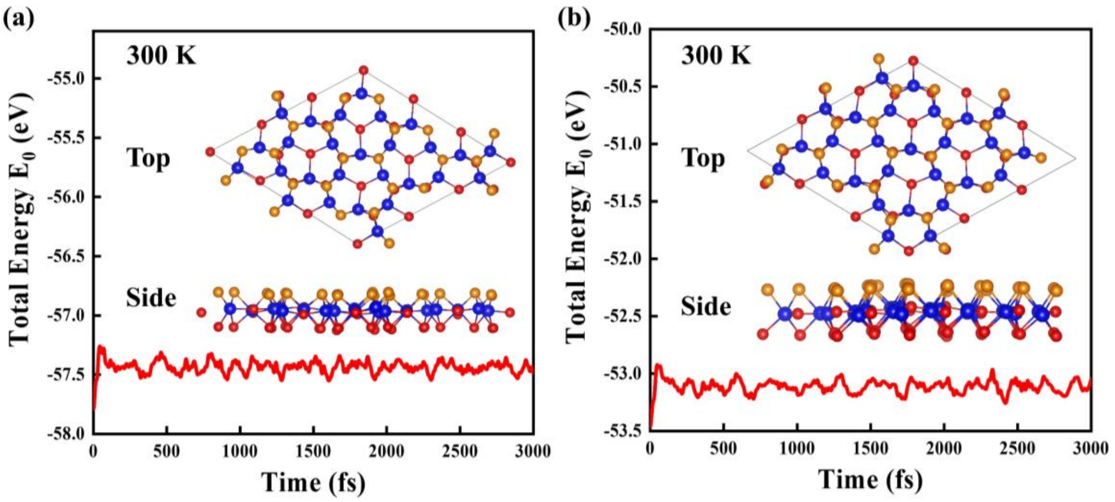

Figure. S2 MD simulation was performed for geometric snapshot of the (a) $V_3Se_3S_2$ and (b) $V_3Se_3Te_2$ monolayer at 300 K for 6 ps.

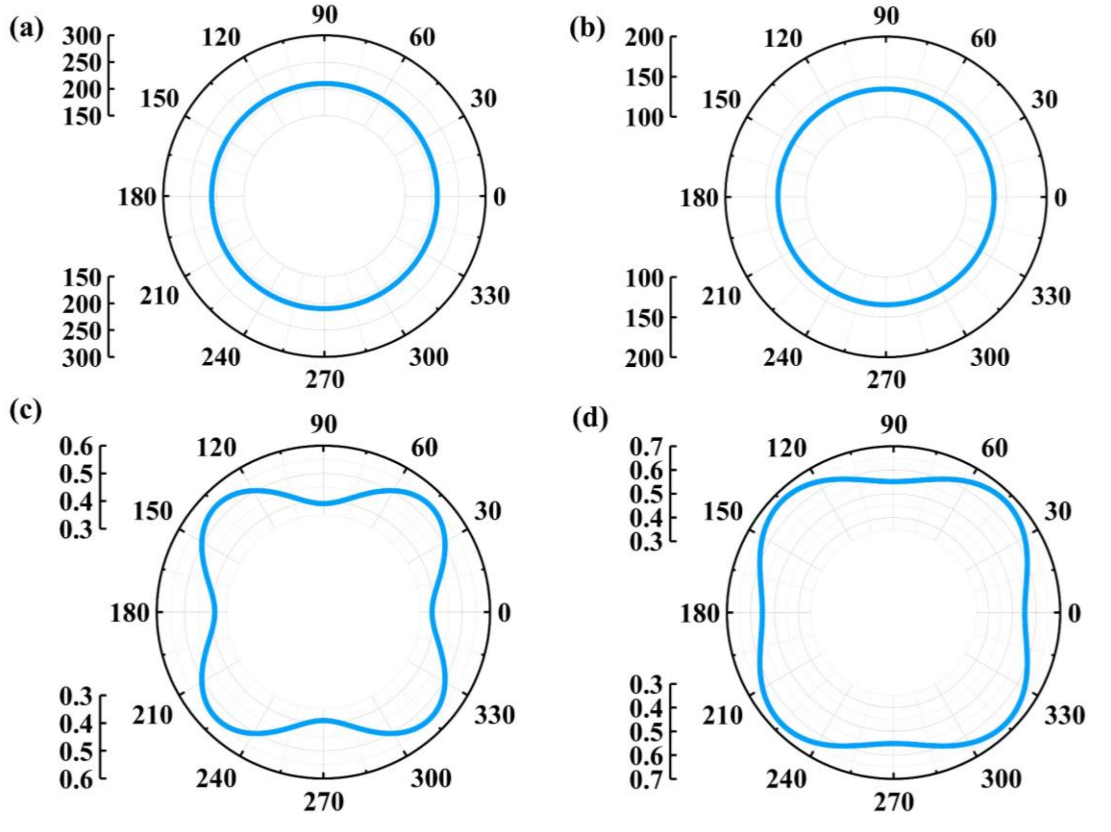

Figure. S3 Young's modules of (a) $V_3Se_3S_2$ and (b) $V_3Se_3Te_2$ respectively and Poisson's ratio of (c) $V_3Se_3S_2$ and (d) $V_3Se_3Te_2$。

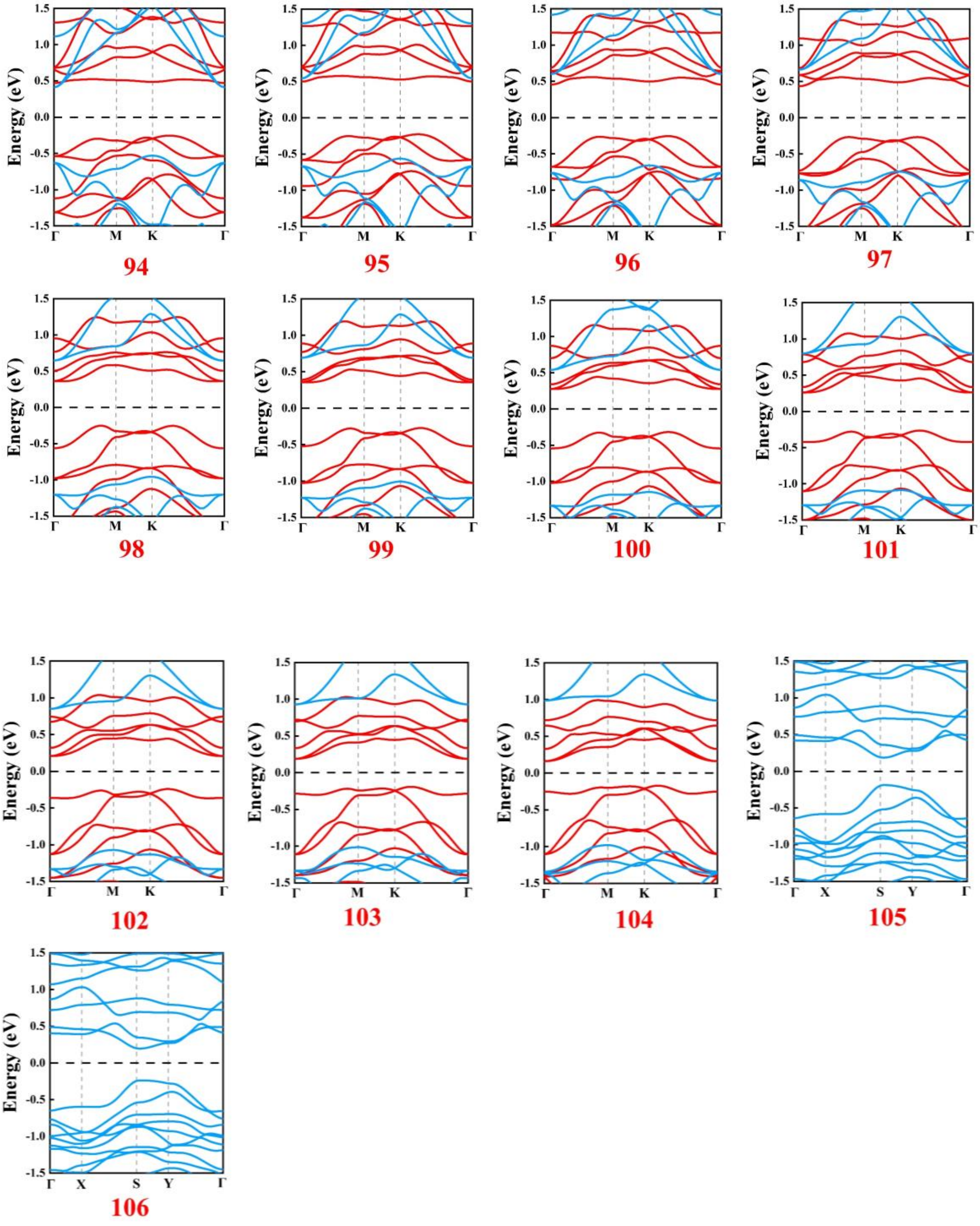

Figure. S4 Band structures of $V_3Se_3S_2$ under biaxial strain from -6% ~ 6%.

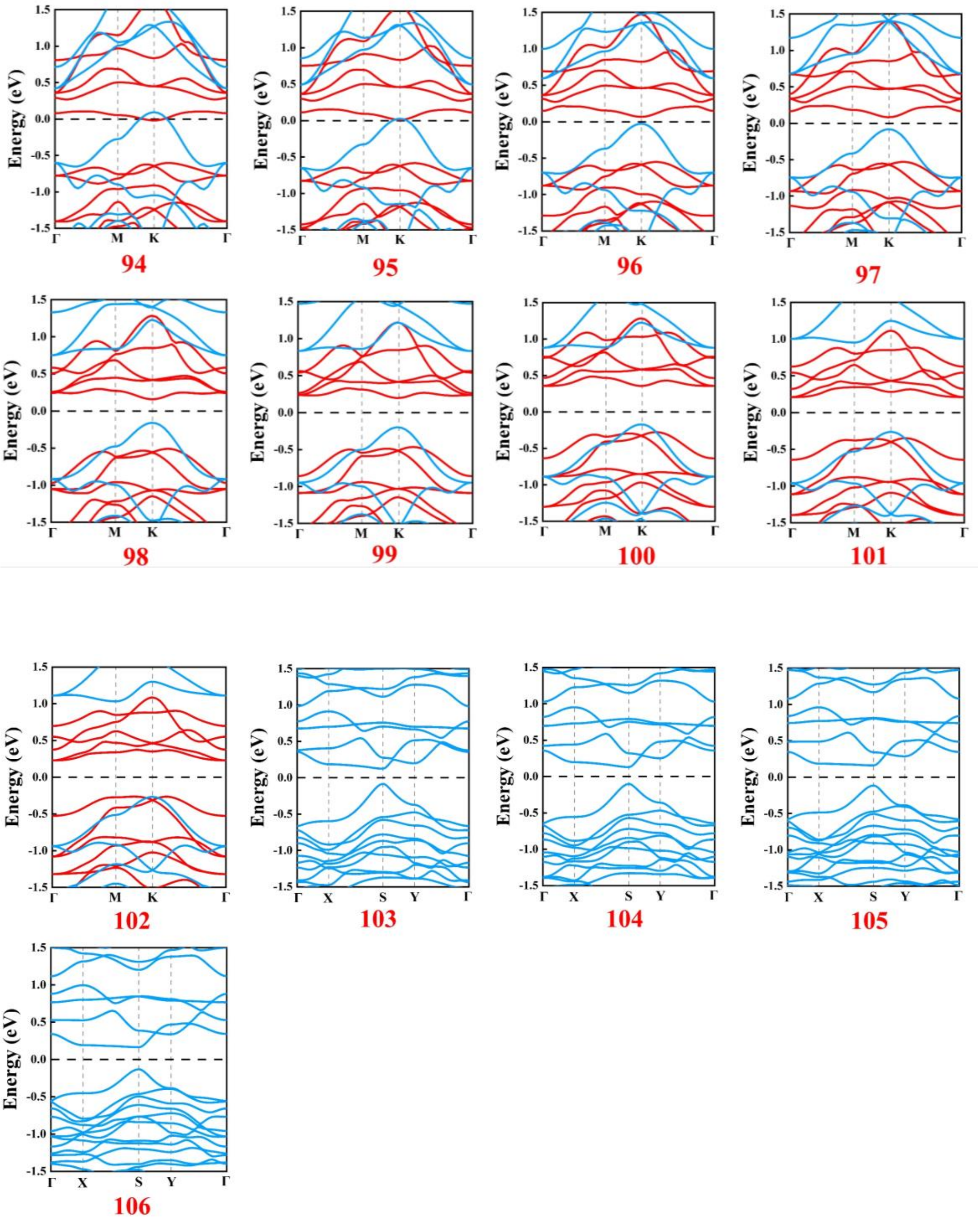

Figure. S5 Band structures of $V_3Se_3Te_2$ under biaxial strain from -6% ~ 6%
